# Proteomic evaluation of genetically modified crops: current status and challenges

**DOI:** 10.3389/fpls.2013.00041

**Published:** 2013-03-07

**Authors:** Chun Yan Gong, Tai Wang

**Affiliations:** Key Laboratory of Plant Molecular Physiology, Institute of Botany, Chinese Academy of SciencesBeijing, China

**Keywords:** genetically modified crops, biological safety, unintended effects, substantial equivalence, proteome and proteomics

## Abstract

Hectares of genetically modified (GM) crops have increased exponentially since 1996, when such crops began to be commercialized. GM biotechnology, together with conventional breeding, has become the main approach to improving agronomic traits of crops. However, people are concerned about the safety of GM crops, especially GM-derived food and feed. Many efforts have been made to evaluate the unintended effects caused by the introduction of exogenous genes. “Omics” techniques have advantages over targeted analysis in evaluating such crops because of their use of high-throughput screening. Proteins are key players in gene function and are directly involved in metabolism and cellular development or have roles as toxins, antinutrients, or allergens, which are essential for human health. Thus, proteomics can be expected to become one of the most useful tools in safety assessment. This review assesses the potential of proteomics in evaluating various GM crops. We further describe the challenges in ensuring homogeneity and sensitivity in detection techniques.

## Introduction

Genetic modification has become the fastest-adopted technology in the history of modern agriculture. It involves the transfer of individual genes that encode specific desirable traits into the host, thus producing genetically modified (GM) crops (also transgenic crops) (Garcia-Canas et al., 2011). GM crops therefore possess improved agronomic traits, such as resistance to insects, tolerance to herbicides, improved productivity and quality, and other traits not present before genetic modification.

The hectares of GM crops continue to greatly increase worldwide (James, 2010; D'Alessandro and Zolla, 2011). Many important crops have been GM by transgenes; examples are maize (*Zea mays*), potato (*Solanum tuberosum*), rice (*Oryza sativa*), wheat (*Triticum aestivum*), soybean (*Glycine max*), tobacco (*Nicotiana tabacum*), tomato (*Solanum lycopersicum*), and cotton (*Gossypium hirsutum*). However, genetic modifications generally represent a double-edged sword. Besides inducing desired traits, modifications in a plant genome might result in unintended effects, which may affect human health or the environment (Ioset et al., 2007). Some unintended effects may result from integration of the transgene and/or biological interactions caused by transgene-encoding proteins, which can be predicted by knowledge of transgene integration sites, transgene function and transgene-related metabolic pathways. However, transgene integration and/or transformation and tissue culture during transgenic progress may induce unintended genomic alterations in GM plants such as deletions, insertions, and rearrangements, which may generate secondary or pleiotropic effects (Kuiper et al., 2001; Cellini et al., 2004; Garcia-Canas et al., 2011). The unintended effects associated with genomic alterations are unpredictable (Garcia-Canas et al., 2011). With the commercialization of GM crops, these unintended effects are one of the most controversial issues in debating the biological safety of GM crops. A systematic comparative analysis of molecular features of GM crops and their comparators is needed to clarify unintended effects (Cellini et al., 2004; Garcia-Canas et al., 2011).

The concept of “substantial equivalence” was proposed as a cornerstone of biological safety assessment in many countries (OECD, 1993). Although “the principle left much scope for individual (and national) interpretation” (Kok and Kuiper, 2003), it is still an acceptable standard to evaluate the biological safety of GM crops. The increasingly use of “omics” technologies, including genomics, proteomics, and metabolomics, in GM crops analysis has provided important information on the molecular characteristics of GM crops and extended our understanding of the biological safety of GM crops. In this mini-review, we briefly discuss technologies used in evaluating GM crops, and summarize current proteomics insights into GM crops.

## Technologies for evaluating GM crops

Targeted analysis is the primary method used for evaluating GM crops. According to the Organisation for Economic Co-operation and Development (OECD, 2006), 50–100 or more analytes are selected for targeted analysis of each crop variety. These analytes typically represent a limited number of crop compositions; they cannot cover unknown toxins, antinutrients, or other secondary products that could result from the genetic modification (Kuiper et al., 2001). Accordingly, targeted analysis is useful for detecting primary or intended but not unintended effects of genetic modification and has been considered biased (Millstone et al., 1999).

Profiling techniques have emerged as useful approaches to evaluate the unintended effects. They allow for simultaneous characterization and comparison of the genome, proteome, and metabolome of an organism, thus increasing the chances of detecting the unintended effects (Kuiper et al., 2003; Ruebelt et al., 2006). These non-targeted approaches have become more comprehensive than targeted analysis in characterizing the composition and performance of GM crops and detecting the unintended effects.

Transcriptome profiling has been used to characterize several GM crops, including maize, barley and rice (Coll et al., 2008, 2009; Kogel et al., 2010; Montero et al., 2011). Transcriptome profiling could be used to investigate gene expression changes in GM crops and has potential to detect unintended effects. But changes in a transcriptome do not necessarily lead to changes in a proteome or metabolome, therefore do not necessarily predict changes in food composition and quality (Chassy, 2010), so transcriptomic profiling is limited in evaluating unintended effects.

Besides transcriptomic techniques, proteomic and metabolomic methods are two complementary tools for evaluating GM crops. In recent years, metabolomic analysis has become prevalent (Catchpole et al., 2005; Baker et al., 2006; Garcia-Villalba et al., 2008; Kim et al., 2009). Because of its ability for use in analyzing a great number of metabolites and reducing the cost of analysis for each analyte, metabolomics has been considered a replacement for conventional compositional analysis (Chassy, 2010). However, this method is only capable of measuring hundreds of metabolites, not the thousands of metabolites in a plant (Davies, 2010). Furthermore, differences in metabolomic methodologies, data analysis, and statistical analysis have resulted in less reproducibility. Therefore, metabolomics may not be advantageous for safety assessment (Chassy, 2010). Proteins are key players in gene function and are directly involved in metabolism and cellular development, thus forming the central bridge between the transcriptome and metabolome (Salekdeh and Komatsu, 2007). Furthermore, proteins have roles as toxins, antinutrients, or allergens, which have great impact on human health. Therefore, proteomic studies would provide important information for understanding changes in biological processes after genetic modification and are important for evaluating biological safety of GM crops.

## Proteomic characteristics of GM crops

2-D gel electrophoresis (2-DE) analysis, established in 1975, revolutionized the study of proteins (Klose, 1975; O'Farrell, 1975) and has helped in the development of proteomics. With use of immobilized pH gradients (Bjellqvist et al., 1982) and mass spectrometry (MS), proteomics has played important roles in understanding the mechanisms and protein–protein interaction networks underlying biological processes. Comparative proteomic strategies combined with 2-DE and MS and with liquid chromatography tandem mass spectrometry (LC-MS/MS) have been extensively used to evaluate the effects of genetic modification on the proteomes of eight GM crops: maize, pea, potato, rice, soybean, tobacco, tomato, and wheat (Table 1). These studies involved safety evaluation of GM crops and functional characterization of GM crops. Some studies investigated different varieties for detecting natural variation. Proteomic evaluation of a GM pea cultivar carrying α-amylase inhibitor-1 (αAI1) was discussed elsewhere (Chen et al., 2009; Islam et al., 2009; Ricroch et al., 2011). An excellent review discussed MS-based identification of transgenic proteins in GM crops (Garcia-Canas et al., 2011). So here we mainly discuss the recent status of proteomic evaluation of the other seven GM crops.

**Table 1:** **Use of proteomic techniques to evaluate genetically modified crops**.

**Crop**	**Donor organism**	**Transgenic protein/*gene***	**Traits**	**Tissues**	**Purpose**	**Proteomic approaches**	**References**
Maize	*Bacillus thuringiensis*	CryIA(b)/*cry*IA(b)	Insect resistance	Seeds	Detection of unintended effects	2-DE, MALDI-TOF MS	Albo et al., 2007
	*Bacillus thuringiensis*	CryIA(b)/*cry*IA(b)	Insect resistance	Seeds	Detection of unintended effects	2-DE, nLC-ESI-IT MS/MS	Zolla et al., 2008
	*Bacillus thuringiensis*	CryIA(b)/*cry*IA(b)	Insect resistance	Seeds	Detection of unintended effects	2-DE, ESI-IT MS/MS	Coll et al., 2011
	*Bacillus thuringiensis*	CryIA(b)/*cry*IA(b)	Insect resistance	Leaves	Detection of unintended effects	2-DE, MALDI-TOF MS	Balsamo et al., 2011
	*Bacillus thuringiensis*	CryIA(b)/*cry*IA(b)	Insect resistance	Seeds	Detection of unintended effects	2-DE, no identification	Barros et al., 2010
	*Agrobacterium tumefaciens*	CP4 EPSPS/*cp4 epsps*	Glyphosate tolerance				
Pea	Bean (*Phaseolus vulgaris*)	α-amylase inhibitor-1/α*ai1*	Pea weevil resistance	Seeds	Detection of unintended effects	DIGE, ESI Q-TOF MS/MS	Islam et al., 2009
	Bean	α-amylase inhibitor-1/α*ai1*	Pea weevil resistance	Seeds	Detection of unintended effects	2-DE, MALDI-TOF MS/MS	Chen et al., 2009
Potato	Potato	Antisense G1-1 gene/*G1-1*	Sprouting delay	Tubers	Functional characterization of GM crops	MALDI-TOF MS	Careri et al., 2003
	*Aureobasidium pullulans*	Glucan branching enzyme/*W2*	Waxy phenotype	Tubers	Detection of unintended effects	2-DE, μLC-ESI-IT MS/MS, μLC-ESI-QqTOF MS/MS	Lehesranta et al., 2005
	Potato	Glycoprotein/*Mal1*	Changes in cell wall structure				
	Potato	AdoMetDC/*SamDC*	Modified metabolism				
	Tomato	Cathepsin D inhibitor/sl*cdi*	n	Leaves	Functional characterization of GM crops	2-DE, SELDI TOF MS	Goulet et al., 2010
	Tomato	Cathepsin D inhibitor/sl*cdi*	n	Tubers	Detection of unintended effects	2-DE, LC-ESI-IT MS/MS	Khalf et al., 2010
Rice	Rice	YK1/YK1	Several stress tolerance	Cultured cells	Functional characterization of GM crops	2-DE, internal amino acid sequencing	Takahashi et al., 2005
	Human (*Homo sapiens*)	hGM-CSF/*hgm-csf*	hGM-CSF reactor	Seeds	Functional characterization of GM crops	iTRAQ, nLC ESI-QTOF MS/MS	Luo et al., 2009
	*Streptomyces hygroscopicus*	PAT/*bar*	Herbicide resistance	Seeds	Detection of unintended effects	DIGE, MALDI-TOF MS	Gong et al., 2012
	*Bacillus thuringiensis*/cowpea	CryIAc/cowpea trypsin inhibitor/*cry1Ac/sck*	Insect resistance	Seeds			
	Sunflower (*Helianthus annuus*)	SSA/SSA	n	Seeds	Functional characterization of GM crops	2-DE, MALDI-TOF MS	Islam et al., 2005
	Rice	thaumatin-like protein/thaumatin-like gene	Bacterial blight disease resistance	Leafs	Functional characterization of GM crops	2-DE, N-terminal amino acid sequencing, MALDI-TOF MS	Mahmood et al., 2009
Soybean	*Agrobacterium tumefaciens*	CP4 EPSPS/*cp4 epsps*	Glyphosate tolerance	Seeds	Optimization of parameters	2-DE, MALDI-QTOF MS	Brandao et al., 2010
	*Agrobacterium tumefaciens*	CP4 EPSPS/*cp4 epsps*	Glyphosate tolerance	Seeds	Functional characterization of GM crops	2-DE, DIGE, MALDI-QTOF MS, MALDI-QTOF MS/MS, ESI-QTOF MS/MS	Barbosa et al., 2012
	*Agrobacterium tumefaciens*	CP4 EPSPS/*cp4 epsps*	Glyphosate tolerance	Seeds	Functional characterization of GM crops	2-DE, LC-MS/MS	Batista et al., 2007
Tobacco	*Arabidopsis thaliana*	AdoMetDC/*AdoMetDC*	Perturbed polyamine metabolism	Leaves	Functional characterization of GM crops	2-DE, MALDI-TOF MS, ESI-QTOF MS/MS	Franceschetti et al., 2004
	Tomato	Prosystemin/Prosystemin gene	Insect resistance	Leaves	Detection of unintended effects	2-DE, MALDI-TOF MS, μLC-ESI-IT MS/MS	Rocco et al., 2008
	n	ScFv B9/scFv B9	Virus resistance	Leaves	Detection of unintended effects	DIGE, MALDI-TOF MS, μLC-ESI-IT MS/MS	Di Carli et al., 2009
Tomato	Tomato spotted wilt virus	TSWV nucleoprotein/*TSWV-N*	Virus resistance	Seeds	Detection of unintended effects	2-DE, MALDI-TOF MS	Corpillo et al., 2004
	n	ScFv G4/scFv G4	Virus resistance	Leaves	Detection of unintended effects	DIGE, MALDI-TOF MS, μLC-ESI-IT MS/MS	Di Carli et al., 2009
	n	ScFv G4/scFv G4	Virus resistance	Leaves	Functional characterization of GM crops	DIGE, μLC-ESI-IT MS/MS	Di Carli et al., 2010
Wheat	Tobacco	Rab-1/*rab*-1	Improved functional properties	Seeds	Detection of unintended effects	2-DE, MALDI-TOF MS, nESI-QqTOF MS/MS	Di Luccia et al., 2005
	*Streptomyces hygroscopicus*	PAT/*bar*	Herbicide resistance	Seeds	Functional characterization of GM crops	2-DE, MALDI-TOF MS, MALDI-IT MS/MS	Horvath-Szanics et al., 2006
	Wheat	LMW-GS/LMW-GS gene	Improved functional properties	Seeds	Functional characterization of GM crops	2-DE, LC-ESI-QTOF MS/MS	Scossa et al., 2008

*n, no information*.

## Maize

GM maize is the second most commercialized GM crop worldwide and has been approved for release in many countries (Coll et al., 2008). MON810 is insect-resistant GM maize produced by inserting a truncated form of the *cry1Ab* gene from *Bacillus thuringiensis* in the maize genome. The proteomes of MON810 varieties and their comparators have been well studied because of their commercial importance (Albo et al., 2007; Zolla et al., 2008; Barros et al., 2010; Coll et al., 2011). Albo et al. (2007) compared the seed proteomes of MON810 variety BT (derived by crossing La73-Bt and La17-Bt) and the near-isogenic control (derived by crossing La73 and La17) and detected differences in six proteins: glucose and ribitol dehydrogenase appeared specific to BT, and endochitinase A to the control, with a difference in expression of triosephosphate isomerase 1, globulin-1 S, cytosolic 3-phosphoglycerate kinase, and aldose reductase between BT and the control. Coll et al. (2011) found a few spots with 1.1- to 1.8-fold change in expression in seed proteomes of two other sets of MON810 varieties, PR33P67, and DKC6575, and their corresponding non-GM controls, PR33P66, and Tietar. The two studies showed that the proteomes of these GM varieties should be virtually identical to that of their comparators. However, Zolla et al. (2008) found that proteomes of seeds from PR33P67 and PR33P66 had 43 differentially expressed proteins (DEPs) possibly caused by the transgene. This situation was probably resulted from different cultured conditions of materials (agriculture field vs. environmentally controlled growth chamber). Barros et al. (2010) evaluated the effect of growing season, growing location, and transgenes on transcriptomes, proteomes, and metabolomes of maize seeds using the GM lines DKC78-15B (hybrid of event MON810) and DKC78-35R (hybrid of event NK603) and the near-isogenic non-GM hybrid variety CRN3505. Transgenes produced less variation in transcript, protein, or metabolite profiles of each sample pair than did environmental factors. Balsamo et al. (2011) compared leaf proteomes of four MON810 varieties and their controls using the same 2-DE-based proteomics and revealed seven DEPs in the DKBYG240–DKB240 pair, five in the DKBYG350–DKB350 pair, and none in the other two GM–non-GM pairs. Thus, the leaf proteomes of four MON810 varieties may have been similar to those of their non-GM counterparts. Together, these data suggested that the expression of transgenes in host maize plants had no significant effects on proteome of the host, and evaluating the molecular characteristics of GM crops should involve environmental factors.

## Potato

Potato is the fourth major crop widely grown worldwide. Despite diverse potato varieties with desired traits, GM potato is still an important topic in potato breeding. Comparative proteomics was used to evaluate variation in tuber proteomes of a large collection of potato genotypes, including different varieties, landraces, and GM lines (Lehesranta et al., 2005). Different varieties and landraces showed clear separations, which indicates extensive genotypic variation; but the differences between GM and non-GM lines were less significant (Lehesranta et al., 2005). A proteomic study by Careri et al. (2003) showed that spatial sites of transgene-encoding proteins affected the proteome of the analyzed sample—GM potato PG50 carrying the antisense *G1-1* gene of unknown function. Phenotypic and expression analysis confirmed that *G1-1* was highly expressed in apical eyes, and its expression regulated transition from dormancy to sprouting tubers (Agrimonti et al., 2000; Marmiroli et al., 2000). A proteomic analysis revealed no difference between PG50 and non-GM lines with use of whole tubers but several differences with use of only apical eyes. This finding was consistent with the expression of *G1-1* gene in apical eyes. Other studies found inconsistent proteome changes in different tissues of GM potato (Goulet et al., 2010; Khalf et al., 2010). Transgenic potato expressing cathepsin D inhibitor (S/CDI) showed significant variation in leaf proteome as compared with the control (Goulet et al., 2010) but no quantitative proteome difference in tubers of the two samples (Khalf et al., 2010). Because antisense *G1-1* in PG50 affects only the proteome of tissue expressing antisense *G1-1*, the different effect of the sl*cdi* transgene on leaf and tuber proteomes may result from different expression of the gene in leaves and tubers, although the two studies did not explore the different expression. Therefore, examining expression patterns and levels of transgenes is important to explain proteomic data.

## Rice

Rice feeds one-quarter of world's population and is one of main crops in the world (Agrawal and Rakwal, 2011). It has become model system to study biological issues. GM rice has been quickly developed although commercialization of GM rice is still in debate. Gong et al. (2012) evaluated proteome differences in seeds from two sets of GM rice (Bar68-1 carrying *bar* and 2036-1a carrying *cry1Ac/sck*) and their controls by 2-DE differential in-gel electrophoresis (2D-DIGE). To obtain relatively objective data, this study included other rice varieties to evaluate proteome variations related to spontaneous genetic variation, genetic breeding, and genetic modifications. GM events did not substantially alter protein profiles as compared with conventional genetic breeding and natural genetic variation (Gong et al., 2012). However, use of GM rice expressing human granulocyte-macrophage colony stimulation factor and shotgun analysis of trypsin digests labeled by isobaric tags for relative and absolute quantitation (iTRAQ) revealed more DEPs (103 proteins) between GM rice and wild-type rice endosperm (Luo et al., 2009). No parallel experiments have compared the output of the two proteomic strategies with the same set of GM rice and control, so explaining these differences is difficult. One explanation may be that the LC-MS/MS shotgun approach provides more comprehensive identification of proteins. In addition, different exogenous genes and/or different insert events of these genes in host genomes may have different impacts on the host crops. Indeed, several proteomic studies of GM rice have investigated biological changes with exogenous gene insertion in seeds (Islam et al., 2005), cultured cells (Takahashi et al., 2005), and leaf blades (Mahmood et al., 2009). A final explanation may be that these transgene-encoding proteins accumulate at different levels in analyzed tissues, for a differential effect on proteome profiling of the analyzed tissues, as we discussed previously for GM potato. Therefore, the safety assessment of GM crops should be case by case.

## Soybean

Soybean is one of the main foods responsible for allergic reactions worldwide, but GM soybean remains one of the most commercialized GM crop. Batista et al. (2007) compared seed proteome of Roundup Ready soybean carrying *cp4 epsps* and non-transgenic control; they found that soybean endogenous allergen expression did not seem to be altered after genetic modification. Brandao et al. (2010) evaluated variables that affect the analysis of protein profiles before comparative proteomic analysis of Roundup Ready soybean (MSOY7575RR) and a non-GM comparator (MSOY7501). Optimal parameters were manual image editing, 300- μg protein loading for 3–10 pH range strips and 500- μg loading for 4–7 pH strips. The authors identified 10 DEPs between seed proteomes via 2-DE with both pH 3–10 and 4–7 gel strips. Barbosa et al. (2012) used a more sensitive 2D-DIGE technique to further compare protein profiles of seeds from the same sample pair and revealed only four DEPs with pH 4–7 strips, of which only one DEP was identical to that identified by Brandao et al. (2010). The two studies used the same materials and protein extraction methods, so the inconsistent results probably derived from standards for judging DEPs in comparative proteomics. Barbosa et al. (2012) identified DEPs based on statistical analysis (*p* ≤ 0.05, by Student *t*-test) and fold change in expression (≥1.5, 50% variation), but Brandao et al. (2010) identified DEPs by only fold change in expression (≥1.8, ~90% variation). Therefore, statistical analysis may be important to determine differential expression of proteins.

## Tobacco

Tobacco is of great economic importance, despite hazards and environmental problems accompanying its production. Di Carli et al. (2009) used 2D-DIGE to compare the leaf proteomes of GM tobacco expressing ScFv (B9) antibody against the G1 envelope glycoprotein of tomato spotted wilt virus (TSWV) and a non-GM control. The recombinant antibody did not significantly affect leaf protein profiles. However, a proteomic study of tobacco expressing tomato prosystemin showed that expression of the gene greatly changed leaf protein profiles of the host (Rocco et al., 2008). The two studies also indicated the need for evaluating substantial equivalence in GM plants on a case-by-case basis. Furthermore, changes in host proteome are associated with the expression level of a transgene, as shown in the study of GM tobacco lines overexpressing S-adenosylmethionine decarboxylase (AdoMetDC) (Franceschetti et al., 2004). A comparison of leaf proteomes from three GM tobacco lines with different levels of AdoMetDC revealed that as compared with the control, the proteome of the GM line with the highest level of AdoMetDC had the largest numbers of DEPs and that of the line with the lowest level of AdoMetD had the lowest numbers of DEPs.

## Tomato

Tomato has great economic and nutritional value. Genetic modifications of tomato mainly involved virus resistance, and delayed fruit ripening. Corpillo et al. (2004) first assessed the substantial equivalence of GM tomato using proteomic approaches. The authors found no qualitative or quantitative differences between the GM tomato that was GM for resistance to TSWV and the non-GM control. Similarly, Di Carli et al. (2009) demonstrated that expression of scFv(G4) against the CMV coat protein in tomato did not cause pleiotropic effects. Di Carli et al. (2010) further evaluated protein profiles of the same scFv(G4)-expressed GM tomato and the wild-type after cucumber mosaic virus (CMV) infection. Proteomic data showed that proteins related to photosynthesis, photorespiration, carbon metabolism, and defense mechanism were downregulated by CMV in infected leaves, which highlighted the mechanisms of the plant–virus interactions.

## Wheat

Wheat is the second most important cereal crop in the world and constitutes a major part of the human diet (Salekdeh and Komatsu, 2007). Currently, GM wheat mainly involves improvement in protein quality. A proteomic study of GM bread wheat overexpressing a low-molecular-weight glutenin subunit (LMW-GS) revealed a series of variations, including overaccumulation of the LMW glutenin and downregulation of all other classes of storage proteins, which constituted a compensatory mechanism (Scossa et al., 2008). The proteomic analysis of two other GM durum wheat, Svevo B730 1-1, expressing the wild-type form of tobacco *rab-1*, and Ofanto B688 1-2, expressing a mutated form of *rab-1*, and their control lines (Svevo and Ofanto) revealed inconsistent results (Di Luccia et al., 2005). Tobacco *rab-1* influences the trafficking of gluten proteins through the secretory system by up- or downregulating the transport step from the endoplasmic reticulum to the Golgi apparatus. Proteomic data showed significant differences between Ofanto B688 1-2, with downregulated trafficking, and the Ofanto control but none between Svevo B730 1-1, with upregulated trafficking, and the Svevo control. The study concluded that the proteomic approach is powerful for investigating protein changes in GM crops and for understanding events involved in quality trait. Indeed, Horvath-Szanics et al. (2006) used proteomic methods to identify stress-induced proteins in herbicide-resistant GM wheat lines and found changed level of LMW seed proteins and sensitivity to drought stress in this GM wheat under drought stress.

## Future and challenges

Proteomics has been used to detect unintended effects caused by genetic modification in GM crops. Results from most studies involving comparative proteomics demonstrated essentially identical protein profiles in GM crops and controls. Furthermore, genetic modification caused less variation in GM crops than the natural variability derived from conventional breeding and genotypic variation. However, some other studies demonstrated that genetic modification affected the proteomes of recipient crops. The discrepancy may be explained by differences in (1) the inserted exogenous genes, (2) the insertion sites of exogenous genes, (3) the expression levels of exogenous genes in recipient crops, (4) the specific organs used for study, (5) GM crop planting conditions, and (6) detection techniques. Therefore, proteomic evaluation of GM crops should be considered on a case-by-case basis.

However, proteomic approaches need improvement. First, homogeneity is needed in experimental quality and data handling (Lay et al., 2006). Experiments should involve as many biological repeats as possible. Statistical analysis and biological validation are also needed. These requirements are applicable to all “omics” techniques. Second, different methods should be combined, including different combinations of “omics” as well as the combination of “omics” and targeted analysis (Ricroch et al., 2011). More sensitive techniques are needed for detecting a larger range of unintended effects.

### Conflict of interest statement

The authors declare that the research was conducted in the absence of any commercial or financial relationships that could be construed as a potential conflict of interest.
